# Production of Nitric Oxide and Expression of Inducible Nitric Oxide Synthase in Ovarian Cystic Tumors

**DOI:** 10.1155/2008/186584

**Published:** 2009-01-05

**Authors:** Rosekeila Simões Nomelini, Lívia Carolina de Abreu Ribeiro, Beatriz Martins Tavares-Murta, Sheila Jorge Adad, Eddie Fernando Candido Murta

**Affiliations:** ^1^Research Institute of Oncology (IPON), Discipline of Gynecology and Obstetrics, Federal University of Triângulo Mineiro, 38025-440 Uberaba, MG, Brazil; ^2^Discipline of Human Anatomy, Federal University of Triângulo Mineiro, 38025-440 Uberaba, MG, Brazil; ^3^Discipline of Pharmacology, Federal University of Triângulo Mineiro, 38015-050 Uberaba, MG, Brazil; ^4^Discipline of Special Pathology, Federal University of Triângulo Mineiro, 38025-440 Uberaba, MG, Brazil

## Abstract

Tumor sections from nonneoplastic (*n* = 15), benign (*n* = 28), and malignant ovarian tumors (*n* = 20) were obtained from 63 women. Immunohistochemistry of the tumor sections demonstrated that inducible nitric oxide synthase (iNOS) expression was increased in ovarian cancer samples compared to nonneoplastic or benign tumor samples. Using the Griess method, nitric oxide (NO) metabolite levels were also found to be elevated in malignant tumor samples compared to benign tumor samples (*P* < .05). For stage I ovarian cancer, intracystic NO levels >80 *μ*M were more frequent than NO levels <80 *μ*M, and iNOS expression in well-differentiated carcinomas was greater than in moderately/poorly differentiated carcinomas (*P* < .05). These data suggest an important role for NO in ovarian carcinogenesis.

## 1. INTRODUCTION

Ovarian cancer is the eighth most frequent malignant neoplasia and the fifth most common cause of death from malignant tumor growth in women in the US [1]. The frequency of ovarian cancer increases in each decade of life, with the highest rate of diagnosis occurring in women that are 75 years of age [2]. Early diagnosis of ovarian tumors is difficult since tumors with diameters less than 5 cm cannot be recognized by bimanual pelvic examination [3, 4]. However, pelvic examination, ultrasonography, detection of tumor markers (such as CA 125, CA 15.3, CA 72.4, and CA 19.9), as well as color Doppler imaging have been shown to be useful in the diagnosis of ovarian cancer, despite the limitations of these methods in differentiating between benign and malignant tumors [5].

Nitric oxide (NO) is a biological messenger synthesized from L-arginine by nitric oxide synthase (NOS). The endothelial and neuronal isoforms of NOS (eNOS and nNOS, resp.) are constitutively expressed in many cell types, however, inducible NOS (iNOS) is only induced in leukocytes, endothelial cells, and other specific cell types after stimulation by bacterial endotoxins or cytokines, resulting in higher concentrations of NO [6].

NO can also exert both pro- and antitumor effects in the tumor microenvironment. Production of NO is a mechanism by which activated endothelium can lyse tumor cells [7], however, it can also regulate tumor growth and metastasis depending on its concentration [8, 9]. Data from previous studies also suggest that NO is both cytotoxic and cytostatic against microorganisms and malignant cells [10, 11] with synthesis of NO by malignant cells causing NO-mediated apoptosis [12]. As a free radical, NO can react to produce peroxynitrites which can directly and indirectly cause DNA damage [12]. If produced for a long period of time, excess NO production can lead to mutations and ultimately to cancer [13, 14]. In addition to increasing the metastatic potential of tumor cells via mutations in the DNA, NO production by neoplastic cells promotes angiogenesis, an essential process for the growth and maintenance of tumors [15, 16].

Understanding the role of iNOS in ovarian cancer would provide valuable insight into the development of additional therapeutic options. The aim of the present study was to identify differences between iNOS expression and the local production of NO in patients with varying stages and grades of ovarian cystic tumors. Therefore, levels of intracystic NO metabolites and expression of iNOS were analyzed in tumor sections from patients with nonneoplastic, benign, or malignant ovarian tumors.

## 2. MATERIALS AND METHODS

### 2.1. Patients and pathological assessment

This study enrolled 63 randomly selected women who received pelvic mass outpatient services from the Discipline of Gynecology and Obstetrics of the Federal University of Triângulo Mineiro (UFTM), Uberaba, Brazil. These patients underwent surgery for an adnexal mass between February 1996 and February 2007, and informed consent was obtained from patients to allow their tissue to be used for examination and related experiments. This study was approved by the UFTM Research Ethics Committee.

Candidates for exploratory surgery were characterized by one or more of the following criteria: cysts with ≥1 thick septum (>3 mm) or ≥2 thin septa, a cyst diameter ≥7 cm, persistence or increase in the cyst or ovarian volume over a minimum of two follow-up periods, the presence of vegetation or calcification, a solid or predominantly solid tumor, ascites, elevated serum levels of tumor markers, or a resistance index ≤0.4 as detected by color Doppler imaging [3, 5]. Inclusion criterion was the anatomicopathological finding of an ovarian tumor (primary neoplastic or nonneoplastic tumor). Exclusion criteria were adnexal torsion, cyst rupture, metastasis of another primary tumor, or previous chemotherapy. The anatomicopathological evaluation and staging of all cases were performed according to guidelines published by the World Health Organization (WHO) and the International Federation of Gynecology and Obstetrics (FIGO) [17, 18]. Patients were divided into 3 groups according to the classification of tumor type: nonneoplastic (*n* = 15), benign (*n* = 28), or malignant (*n* = 20) (which included cystadenomas of borderline malignancy). Characterization of patient groups is presented in Table 1.

### 2.2. Collection of intracystic fluid

Cystic fluid samples were aseptically collected by punction immediately following resection of tissue. Bloody fluids caused by punction were excluded from analysis. The collected fluids were immediately stored on ice until centrifugation (180× g, 15 minutes) was performed. Cell supernatants were transferred to fresh tubes, and the cell pellets were resuspended in phosphate-buffered saline (PBS). Both samples were stored at −70°C until needed [19].

### 2.3. Determination of nitrate concentration

The levels of NO metabolites (nitrite plus nitrate) in cystic samples were determined by enzymatically reducing the nitrate present with nitrate reductase as previously described [20]. Briefly, 50  *μ*L of nondiluted sample was incubated with an equal volume of reductase buffer (0.1 M potassium phosphate (pH 7.5), 1 mM NADPH, 10 mM FAD, 4U of nitrate reductase/mL) for 20 hours at 37°C. A standard nitrate curve was obtained by incubating sodium nitrate (10 to 200  *μ*M) with reductase buffer. The total amount of nitrite was determined by the Griess method [21]. Briefly, the samples were incubated with an equal volume of freshly prepared Griess reagent (1% sulfanilamide, 0.1% naphthylenediamine dihydrochloride in 5% phosphoric acid). Absorbance at 550 nm was determined using a multi-well plate reader (Multiskan MCC/340MKII, Flow Laboratories) and the results were reported as micromoles of NO_3_ + NO_2_.

### 2.4. Immunohistochemistry

Specimens obtained from surgical resection were fixed in 10% formalin before being processed in paraffin. Sections stained with hematoxylin-eosin were reviewed by a pathologist and a representative section for each case was selected for immunohistochemical analysis.

Selected sections were deparaffinized, rehydrated, and heated in a microwave oven in 0.01 M citrate buffer (pH 6.0) for 30 minutes. Endogenous peroxidase activity was blocked by 3% hydrogen peroxide for 10 minutes, followed by a wash with PBS. The sections were incubated overnight at 4°C with an anti-iNOS rabbit polyclonal antibody (Santa Cruz Biotechnology, 1:200). The conjugate used was the avidin-biotin peroxidase detection solution (Dako Cytomation LSAB and System-HRP). The signal was visualized using diaminobenzidine (Dako Cytomation Liquid DAB and substrate Chromogen System, Dako). Slides were counterstained with Harris's haematoxylin, dehydrated, cleared, and mounted. A skin sample with chronic granulomatous inflammation known to be positive for iNOS was used as a positive control. Two independent observers evaluated the sections and the intensity of staining was evaluated subjectively using the following designations: 0 (no signal), 1 (weak), 2 (medium), 3 (strong) [22]. When scores of multiple tissue stainings were combined, scores that were ≤1 were labeled “weak intensity”, and scores ≥2 were labeled “strong intensity”.

### 2.5. Statistical analysis

Data were analyzed using GraphPad Instat software. For immunohistochemical staining, the concordance between staining intensity scores was calculated according to the following classifications: kappa <0.4: slight concordance; kappa ≥0.4 and <0.8: moderate concordance; kappa ≥0.8 and <1: strong concordance; kappa = 1: perfect concordance. The first inter-rater agreement was 90.7% (kappa = 0.94). All discordant cases were re-evaluated and the result determined by consensus. The Fisher's exact test was used to compare iNOS immunohistochemistry results and to assess the relationship between iNOS expression and NO levels with the stage and grade of the ovarian cancer samples analyzed. Data for nitrate levels were expressed as the mean +/− standard deviation (SD) and values were compared by ANOVA followed by the Tukey test for individual comparisons. The correlation between intracystic nitrate levels and iNOS immunolabeling was tested using Spearman's rank correlation coefficient. The significance level was set at less than 0.05.

## 3. RESULTS

### 3.1. Patients

Sixty-three randomly selected women receiving pelvic mass outpatient services from the Discipline of Gynecology and Obstetrics of the Federal University of Triângulo Mineiro (UFTM) were enrolled in this study. For those diagnosed with nonneoplastic tumors (*n* = 15), 10 (66.7%) had serous ovarian cysts and 5 (33.3%) had functional cysts (corpus luteum, follicular, and theca lutein cysts). The 28 patients diagnosed with benign neoplasias included 11 (39.3%) with serous cystadenoma, 6 (21.4%) with mucinous cystadenoma, 6 (21.4%) with mature teratoma, 3 (10.7%) with cystadenofibroma, as well as 1 (3.6%) with serous- and 1 (3.6%) with mucinous-cystadenoma associated with Brenner's tumor. For the 20 patients diagnosed with malignant neoplasias, the cases included 7 (35%) serous adenocarcinoma, 3 (15%) granulosa cell tumor, 3 (15%) mucinous cystadenoma of borderline malignancy, 2 (10%) mucinous cystadenocarcinoma, 1 (5%) endometrioid adenocarcinoma, 1 (5%) granulosa cell tumor associated with Brenner's tumor, 1 (5%) anaplasic adenocarcinoma, 1 (5%) immature teratoma with epidermoid carcinoma, and 1 (5%) serous cystadenoma of borderline malignancy. In the ovarian cancer patient group, the number of each tumor stage resected included 5 (25%) of I-A, 1 (5%) of I-B, 2 (10%) of I-C, 1 (5%) of III-A, 10 (50%) of III-C, and 1 (5%) of IV. In the III-C and IV stages, five complete surgeries, four satisfactory citorreductions and only 2 unsatisfactory citorreductions were performed. Patients receiving adjuvant first-line chemotherapy received either a combination of cisplatin, epirubicin, and cyclophosphamide for epithelial tumors, or cisplatin, etoposide, and bleomycin for granulosa cell tumors of stages ≥I-B. Of the 12 carcinomas, 5 (41.7%) were well differentiated, 4 (33.3%) were moderately differentiated, and 3 (25%) were poorly differentiated. Five patients were diagnosed with malignant ovarian tumors had died before their follow-up.

### 3.2. iNOS immunohistochemistry

**Figure 1:**
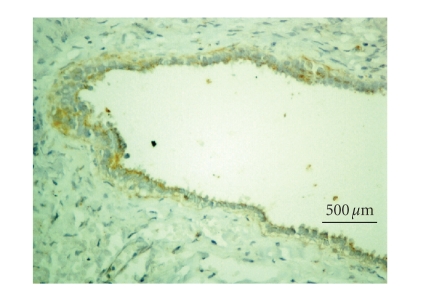
Immunohistochemistry negative staining of anti-iNOS polyclonal antibody (serous ovarian cyst, 400x, diaminobenzidine).

**Figure 2:**
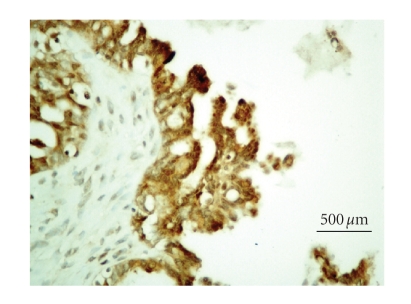
Immunohistochemistry positive staining of anti-iNOS polyclonal antibody (serous cystadenocarcinoma, 400x, diaminobenzidine).

There were sufficient samples to perform iNOS immunohistochemistry on 15 nonneoplastic tumors, 21 benign tumors, and 18 malignant tumors (Figures 1 and 2). The results are summarized in Table 2. Samples with strong iNOS staining (scored ≥2) were more frequently found in ovarian cancer samples than in nonneoplastic (*P* = .0014) or benign neoplasia samples (*P* = .0003).

Samples of malignant ovarian cancer tissue were further classified into well-differentiated (*n* = 11) or moderately/poorly differentiated (*n* = 7) tumors. For the well-differentiated tumors, 8 (72.7%) presented strong staining of iNOS while the other 3 presented weak or medium staining of iNOS. For the moderately/poorly differentiated tumor samples, all showed weak/medium intensity of iNOS. Overall, well-differentiated tumors presented a higher frequency of strong iNOS expression compared to moderately/poorly differentiated carcinomas (*P* = .004; Fisher's exact test). No statistically significant correlation was found between the intensity of iNOS staining and tumor stage.

Of the 5 (27.8%) patients diagnosed with malignant ovarian tumors that had died by the time of follow-up, only one of the samples previously collected from those five individuals showed strong expression for iNOS.

### 3.3. Cystic fluid nitrate concentration

**Figure 3:**
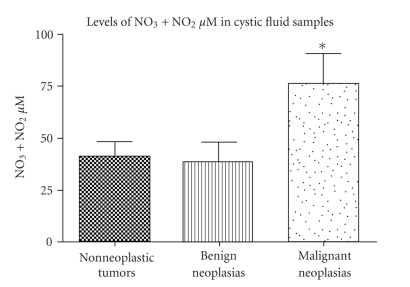
NO metabolite levels determined from cystic fluid samples obtained from patients with nonneoplastic tumors (*n* = 14), benign tumors (*n* = 27), and malignant neoplasias (*n* = 18). *P* = .045 (ANOVA); *P < .05 versus benign neoplasia (Tukey).

Cystic fluid samples were collected at the time of surgical resection and were subsequently tested for NO metabolite levels. Cystic fluids from 1 nonneoplastic tumor, 1 benign neoplasia, and 2 malignant ovarian tumors were not tested due to the viscous consistency of those fluids. The mean levels of NO metabolites detected in the malignant tumor samples (75.7 *μ*M, *n* = 18) were significantly higher (*P* = .045) than the NO metabolite levels for benign ovarian tumors (38.5 *μ*M, *n* = 27). However, statistically significant differences were not detected between NO levels of malignant neoplasia samples versus nonneoplastic samples (40.9 *μ*M, *n* = 14) (Figure 3).

To examine whether intracystic NO metabolite levels could be predictive for tumor stage, patient samples were divided into two groups: those with NO metabolite levels <80 *μ*M and those with NO metabolite levels >80 *μ*M. The value of 80 *μ*M was derived from the median value of NO detected in malignant tumor samples (81.6 *μ*M). No stage II samples had detectable levels of NO metabolites. For stage I samples, 6 (85.7%) had NO metabolite levels >80 *μ*M, and only 1 (14.3%) had NO metabolite levels <80 *μ*M. In contrast, 3 of stage III/IV tumor samples (27.3%) had NO metabolite levels >80 *μ*M and 8 (72.7%) had NO metabolite levels <80 *μ*M. These results indicate that intracystic NO metabolite levels >80 *μ*M were significantly more frequent in stage I samples than in stage III/IV samples (*P* = .0498; Fisher exact test). However, there was no significant correlation between NO metabolite levels and the grade of tumor differentiation.

### 3.4. iNOS immunoreactivity and NO metabolite levels

Correlations between NO production and iNOS expression in the collected tumor tissues are summarized in Table 3. Cystic levels of NO did not correlate with iNOS expression in ovarian tissues.

## 4. DISCUSSION

Differences in iNOS expression between nonneoplastic, benign and malignant ovarian neoplasias suggest a role for NO in ovarian carcinogenesis. Our experiments revealed greater expression of iNOS in malignant ovarian neoplasias than in benign or nonneoplastic tumors. There were two cases of ovarian cancer that did not show iNOS immunoreactivity, while approximately one-third of benign and nonneoplastic tumor samples were positive for iNOS expression. Our results are consistent with other studies that have shown that a majority of ovarian malignant neoplasias present NOS activity, while iNOS is detected at lower levels in patients without cancer [23]. Although we hypothesize that iNOS may be a marker to detect malignant disease, it is not considered by others to be a marker exclusive to malignant disease [24].

Expression of NOS in malignant tissue derived from gynecological, breast, central nervous system, gastric, and colorectal tumors has been reported, suggesting its role in cancer progression [25–29]. A positive correlation between iNOS expression and an increased density of tumor microvessels in human colorectal cancer was shown by Cianchi et al. [28], and iNOS expression has been associated with increased vascularization and tumor invasion in endometrial malignant neoplasia [30]. Correspondingly, patients with lung cancer, prostate cancer, or cervical cancer treated with NO inhibitors showed antivascular activity [31]. These data support the hypothesis that the inhibition of iNOS may provide a new therapeutic option for the treatment of ovarian cancer [32].

Recently, it was demonstrated that iNOS expression in serous and low-grade carcinomas was significantly higher than in nonserous and high-grade carcinomas [22]. Advanced stage tumors expressed low levels of iNOS and were associated with a shorter mean survival time although this was not determined to be a statistically significant correlation. In our study, patients with malignant tumors had significantly higher levels of intracystic NO and iNOS expression in well-differentiated carcinomas compared to moderately/poorly differentiated carcinomas. In addition, positive iNOS expression in ovarian carcinoma had been identified as a positive disease-related survival indicator [33], and was found to be consistent with the high levels of iNOS activity and NO production of nonmetastatic cells versus metastatic cells [34].

In contrast to our findings, a separate study found that patients with advanced ovarian serous tumors express iNOS and COX-2 and experience a shorter disease-free interval and survival rate [35]. In addition, patients negative for iNOS expression presented a complete clinical response to a first-line treatment of chemotherapy. A separate study found a positive correlation of NO synthesis with tumor progression in a breast cancer model [25].

In a study by Taveres-Murta et al., increased levels of NO metabolites in the tumor microenvironment were found in patients with ovarian cancer, but not in patients with benign neoplasia [19]. Similarly, supernatants of cell cultures obtained from well-differentiated, malignant ovarian tumors were found to contain higher levels of NO metabolites compared to cell cultures from patients with poorly differentiated tumors [36]. However, to our knowledge, this is the first study to evaluate both intracystic NO production and iNOS expression from the same tumor tissue. Our results suggest that intracystic NO is not produced by tumor cells since no significant correlation was found between the levels of NO metabolites detected and the intensity of iNOS expression detected by immunohistochemical staining of the tumor tissue. Instead, an analysis of NO production from effusions (ascitic, cystic, or pleural fluids) of ovarian malignant tumors showed a significant correlation between the percentage of macrophages present and detectable levels of NO metabolites, suggesting that macrophages play a significant role in the production of NO in the tumor microenvironment [36].

An association between increased intracystic leukocyte infiltrates and NO production has previously been associated with ovarian cancer [19]. NO produced by ovarian carcinoma cell lines has also been shown to correlate with the extent of tumor cell apoptosis observed [12]. These results, in combination with our data, suggest that the level of iNOS expressed by tumor cells and infiltrating leukocytes accounts for the intracystic NO production detected although it cannot be ruled out that the iNOS associated with ovarian tumors could also be expressed by immune cells [36, 37]. Studies using activated macrophages treated with NOS-specific inhibitors have shown an inhibition of NO production and induced cytotoxicity, suggesting that activated macrophages may mediate NO-dependent cytotoxicity [13].

High concentrations of NO can mediate cytotoxic activity against tumor cells, while low concentrations have been associated with angiogenesis. High levels of NO have also been shown to induce apoptosis. Correspondingly, high concentrations of NO have not been found to be maintained in many malignant neoplasias [38]. These opposing actions of NO have been attributed to factors such as differences in the isoform of NOS expressed, the level of NOS expression, and the type of cell involved in either in vitro or in vivo systems [39].

In ovarian cancer cell lines, high levels of NO donors or strong expression of the iNOS suppressed survivin levels (a human gene that is part of the inhibitor of apoptosis family), and have been shown to induce apoptosis. It is hypothesized that NO signaling could contribute to therapy resistance in epithelial ovarian cancer by modulating survivin expression since low levels of NO are associated with resistance to carboplatin- and paclitaxel-induced apoptosis [40]. The ability to modulate NOS gene expression may represent an opportunity to control the growth and metastasis of tumors in vivo [13].

## 5. CONCLUSIONS

In this study, increased expression of iNOS and increased production of NO metabolites were detected in the tumor microenvironment of ovarian cancer samples compared to benign and nonneoplastic ovarian tissue samples. For ovarian cancer samples, iNOS expression correlated with the extent of tumor differentiation and intracystic NO metabolite levels correlated with the tumor stage. We hypothesize that the absence of a correlation between NO production and iNOS expression indicates that different cell types are involved in iNOS expression, and that controlling NO production and inducing NOS may represent valuable strategies in the prevention of benign tissue transitioning into well-differentiated malignant ovarian tumors.

## Figures and Tables

**Table 1:** Characteristics of the three-patient groups compared in this study.

	Nonneoplastic tumors (*n* = 15)	Benign neoplasias (*n* = 28)	Malignant neoplasias (*n* = 20)
Age (years, mean ± SD)	42.8 ± 7.0	37.1 ± 14.6	46.9 ± 14.9
Parity child (mean ± SD)	2.3 ± 1.5	2 ± 1.8	3.1 ± 2.8
Smokers	4 (26.7%)	7 (25.0%)	9 (45.0%)
Race:			
* *Caucasian women	12 (80.0%)	17 (60.7%)	17 (85.0%)
* *Non-Caucasian women	3 (20.0%)	11 (39.3%)	3 (15.0%)
Use of hormonal			
* *contraception	2 (13.3%)	5 (17.9%)	2 (10.0%)
Tubal ligation	3 (20.0%)	3 (10.7%)	4 (20.0%)
Hormonal status:			
* *Reproductive age (years)	12 (80.0%)	23 (82.1%)	10 (50.0%)
* *In menopause	3 (20.0%)	5 (17.9%)	10 (50.0%)

**Table 2:** Immunohistochemical staining of iNOS in nonneoplastic, benign, and malignant ovarian tissue samples.

	Strong intensity of iNOS expression^(a)^	Weak intensity of iNOS expression^(b)^
Nonneoplastic tumors (*n* = 15)	5 (33.3%)	10 (66.7%)
Benign neoplasia (*n* = 21)	6 (28.6%)	15 (71.4%)
Malignant neoplasia (*n* = 18)*^+^	16 (88.9%)	2 (11.1%)

^(a)^ Received a score of 2-3 for intensity of iNOS expression.

^(b)^ Received a score of 0-1 for intensity of iNOS expression.

*P = .0014 compared to nonneoplastic tumor samples.

^+^ *P* = .0003 compared to benign neoplasia samples (Fisher's exact test).

**Table 3:** Correlation between iNOS immunostaining and NO metabolite levels detected in tissue samples collected using Spearman's rank correlation coefficient.

Groups analyzed	iNOS immunostaining × NO metabolite levels	
Nonneoplastic tumors (*n* = 14)	*r* = 0.1595, *P* = .586	
Benign neoplasia (*n* = 20)	*r* = 0.0942, *P* = .693	
Malignant neoplasia (*n* = 16)	*r* = 0.1188, *P* = .661	
